# Virtual simulation in healthcare education: a multi-professional, pan-Canadian evaluation

**DOI:** 10.1186/s41077-023-00276-x

**Published:** 2024-01-10

**Authors:** Margaret Verkuyl, Efrem Violato, Nicole Harder, Theresa Southam, Mélanie Lavoie-Tremblay, Sandra Goldsworthy, Wendy Ellis, Suzanne H. Campbell, Lynda Atack

**Affiliations:** 1 School of Community and Health Studies, Centennial College, P.O. Box 631, Station A, Toronto, Ontario M1K 5E9 Canada; 2 Centre for Advanced Medical Simulation, Northern Alberta Institute of Technology, 11762 - 106 Street, Edmonton, Alberta T5G 2R1 Canada; 3 College of Nursing, University of Manitoba, 89 Curry Place, Winnipeg, Manitoba R3T 2N2 Canada; 4 Teaching and Learning Centre, Selkirk College, 301 Frank Beinder Way, Castlegar, British Columbia V1N 4L3 Canada; 5 Faculty of Nursing, University of Montreal, Pavillon Marguerite-d’Youville, 2375, chemin de la Côte Sainte-Catherine, Bureau 2089, Montréal, QC H3T 1A8 Canada; 6 School of Nursing and Midwifery, Mount Royal University, 4925, Mount Royal Gate SW, Calgary, Alberta T3E 6K6 Canada; 7 Sally Horsfall Eaton School of Nursing, George Brown College, 51 Dockside Drive, Toronto, Ontario M5A 1B6 Canada; 8 School of Nursing, The University of British Columbia, T201-2211, Wesbrook Mall, Vancouver, British Columbia V6T 2B5 Canada

**Keywords:** Virtual simulation, Simulation pedagogy, Post-secondary education, Clinical practice

## Abstract

**Background:**

As we experience a shortage of healthcare providers in Canada, it has become increasingly challenging for healthcare educators to secure quality clinical placements. We evaluated the impact of virtual simulations created for the virtual work-integrated learning (Virtu-WIL) program, a pan-Canadian project designed to develop, test, and offer virtual simulations to enrich healthcare clinical education in Canada. Evaluation was important since the virtual simulations are freely available through creative commons licensing, to the global healthcare community.

**Methods:**

Students self-reported their experiences with the virtual simulations and the impact on their readiness for practice using a survey that included validated subscales. Open-ended items were included to provide insight into the students’ experiences.

**Results:**

The evaluation included 1715 Nursing, Paramedicine and Medical Laboratory students enrolled in the Virtu-WIL program from 18 post-secondary universities, colleges, and institutions. Results showed most students found the virtual simulations engaging helped them learn and prepare for clinical practice. A key finding was that it is not sufficient to simply add virtual simulations to curriculum, careful planning and applying simulation pedagogy are essential.

**Conclusion:**

Virtual simulation experiences are increasingly being used in healthcare education. Results from this rigorous, large-scale evaluation identified ways to enhance the quality of these experiences to increase learning and to potentially decrease the number of hours healthcare students need in clinical practice to meet professional competencies. Further research is needed regarding many aspects of virtual simulations and, in particular, curriculum integration and the timing or sequencing of virtual simulations to best prepare students for practice.

**Supplementary Information:**

The online version contains supplementary material available at 10.1186/s41077-023-00276-x.

## Highlights

• We investigated the effectiveness of virtual simulation in post-secondary healthcare education

• This is one of the first evaluations to include a national sample of post-secondary students and virtual simulation.

• Virtual simulation, when sound pedagogy and curriculum planning are applied, can be an engaging and effective way for students to prepare for clinical practice.

**Supplementary Information:**

The online version contains supplementary material available at 10.1186/s41077-023-00276-x.

## Background

In 2021, Colleges and Institutes Canada, a national association representing the Canadian publicly funded college system, in collaboration with Simulation Canada, an interprofessional non-profit network for advancing simulation in education and healthcare, led the development of a virtual work-integrated learning (Virtu-WIL) program. The program was funded by the Canadian government with more than eighty stakeholders contributing to the project from 24 colleges and institutions and 13 universities. The goal of the project was to develop, test, and offer virtual simulations (VSs) to support post-secondary healthcare education in Canada. The VSs are openly licensed by creative commons for Canadian educators and students, and many are accessible to educators globally by enrolling at https://simulationcanada.ca/virtu-wil/.

The driving force for the program was the ever-increasing challenge of providing quality work-integrated learning experiences for students, a situation which became markedly difficult during the COVID-19 pandemic [1]. Work-integrated learning opportunities are increasingly being sought in post-secondary institutions as these experiences help students develop employability skills. In a study of over 3000 students, Smith et al. [2] found that by deliberately integrating theory with practice, work-integrated learning can help students engage in self-development, become proficient and ethical practitioners, use information wisely, collaborate with each other and apply theory in novel situations. Because quality clinical placements are difficult to find, educators are interested in exploring innovations in work-integrated learning [3] with VSs being a rapidly emerging method for providing realistic work experiences to healthcare students [4].

Virtual simulations are an interactive learning process where students use screen-based platforms/software that portray realistic clinical events [5]. They offer many unique teaching and learning advantages; students are exposed to realistic, interactive learning experiences, often receiving immediate feedback, which is critically important to learning [6, 7]. Virtual simulations are a flexible teaching and learning approach that provide an environment where students can safely develop clinical skills and repeat patient cases as often as needed [8]. Other drivers for the Virtu-WIL program are the need to better prepare students for clinical placements and to make segments of the curriculum, where students typically struggle, more interactive [9].

The Virtu-WIL VSs are screen-based and designed to be accessed from a computer, tablet, or smart-phone. Screen-based simulation refers to “a simulation presented on a computer screen using graphical images and text, similar to popular gaming formats, where the operator interacts with the interface using keyboard, mouse, joystick, or other input device” ([10], p. 43). The development teams included faculty who were trained in VS development by Virtu-WIL. Seven simulation platforms were used to develop the simulations such as Affinity, PCS, and Body Interact. These platforms are designed to provide simulations in which the learner has to make clinical decisions related to a patient scenario through branching scenarios or interactive options accessed through a mouse click or natural language. The VSs were designed to build students’ technical skills for clinical practice, however, communication, time management, and relationship-building skills were integral and activities to encourage problem-solving and critical thinking regarding patient assessment and care were embedded in the VSs. The Colleges and Institutes Canada diversity and inclusion committee also reviewed the VSs and advised the development teams regarding the content. On completion, the VSs were peer-reviewed and pilot-tested with students.

Initially, approximately 71 Nursing (NUR), 19 Medical Laboratory Technologist (MLT), and 13 Paramedic (PM) VSs were developed (75% English, 25% French). The first intake of students was enrolled in January 2022 across Canada and a second intake was enrolled in September 2022 to February 2023. Students were recruited through an open call sent by their institutional program coordinator or through specific courses. Schools offered the Virtu-WIL program to students in different programs which means that some students, such as those from the internationally educated nursing or bridging from registered practical nurse to registered nurse programs had practiced as professionals in clinical practice. In addition, the Virtu-WIL program was offered to students in different years of the program, as a result, some students had been in clinical practice and others had not. Interested students were directed by educators to Outcome Campus Connect to create accounts and enroll in the Virtu-WIL program. Three healthcare programs participated: NUR, PM, and MLT. Students were expected to complete three to five VSs and attend a debrief either after each VS or after all were completed. Students were offered an incentive for participating in the project.

Faculty and VS facilitators were encouraged, but not required to embed the VSs in the curriculum and all followed a pedagogical process that included a prebrief and a debrief. Given the national scope of the project, there was variable use of the VSs, with some institutions embedding the VSs in the curriculum, and other institutions using the VSs as a standalone activity. Each facilitator was provided with a prebrief template that could be adjusted related to the VS experience. The prebrief template included learning objectives, timelines, fiction contract, confidentiality statement, suggested ground rules, and psychological safety considerations. Sample wording to enhance psychological safety during the debrief and semi-structured debriefing questions based on a debriefing framework were provided with each VS. Facilitators could use these or create their own question based on a different debriefing framework. A clinical expert or industry partner often joined the facilitator for the debrief, they were from the same profession as the learners. The most common types of debrief used were virtual facilitated synchronous debriefing and in-person facilitated group debrief, although self-debriefing was also used. All facilitators had access to professional development related to simulation pedagogy and a subsequent workshop was launched to further develop skills, recognizing the importance of formal training [5, 11].

An evaluation was conducted to measure the Virtu-WIL program’s effectiveness with a view to improving the program. Specifically, we were interested in evaluating the program’s impact on healthcare students’ learning, competency development, and readiness for the workplace. A pan-Canadian working group consisting of educators, teaching, and learning specialists and researchers was established to develop the evaluation framework, guide the evaluation process, and interpret results. This paper focuses on the survey component of the evaluation.

Working with an advisory committee from CICan, the evaluation team developed the following evaluation questions:

1. Is the Virtu-WIL program an innovative and effective WIL model?
2. Does the Virtu-WIL increase learner readiness for practice?
3. Does the Virtu-WIL program promote learning?
4. Does the Virtu-WIL program promote learner competency?

A matrix outlining outcomes, participants, methods, tools, and data analysis was developed to guide evaluation activities.

## Ethics

As the goal of the project was quality improvement, the Tri-council Policy Statement on the Ethical Conduct of Research, which governs research ethics in Canada, made the survey component of the project exempt from ethics review. Any student who wished to obtain the project incentive was required to provide contact information; however, survey completion was entirely voluntary. When completing the evaluation survey, participants were advised of the purpose of the evaluation and that data collected would be kept strictly confidential. Participants were advised that no individuals would be identified when reporting and data would only be reported in aggregate form.

### Sample

The evaluation included all students enrolled in the Virtu-WIL program from three programs: NUR, PM, and MLT in universities and colleges across Canada. The data was collected between October 2022 and April 2023.

## Methods

A survey was developed to evaluate students’ experiences with the Virtu-WIL program and the impact of Virtu-WIL on student readiness for the labour market. This survey, the Exit Survey, was pilot-tested with 181 participants from six schools. Item analysis indicated the survey was functioning adequately and minor revisions were made to some demographic items. The Exit Survey was distributed by email link with the survey hosted online through “Hosted in Canada.” The survey included 26 items clustered under four domains: *overall experience with the Virtu-WIL program*, *virtual simulation player experience*, *debriefing experience*, and *perceived impact of the VS on clinical practice*. To measure the virtual simulation player experience and the debriefing experience, two existing tools that demonstrate good validity evidence were used. These were the Player Experience Inventory (mini-PXI) short form and the Debriefing Experience Scale.

The Player Experience Inventory (mini-PXI) items were added to the Exit Survey as the items provide insight into the student’s experience with VS. Specifically, the items evaluate players’ perceptions of the functional and psychosocial consequences of virtual game play [12, 13]. Functional consequences include five constructs: ease of control, progress feedback, audiovisual appeal, goals and rules, and challenge. Psychosocial consequences include five outcomes: a sense of mastery, curiosity, immersion, autonomy, and meaning. The mini-PXI has undergone rigorous testing including seven research studies to test reliability and validity of the mini-PXI’s items [14] and had been used previously by members of the evaluation working group [15]. The PXI demonstrates adequate reliability for each construct assessed, ranging from .61 to .87, with the mini-PXI’s single-item measures showing strong to moderate correlations with the respective PXI constructs [16]. Two items, separate from the mini-PXI, were added to the broader survey to understand the unique components of gameplay related to prebriefing and clinical practice.

The second set of items added to the Exit survey were items from one subscale of the Debriefing Experience Scale (DES) developed by Reed [17]. The items are used to measure students’ experiences after their assigned debrief. This subscale was added to the Exit Survey as debriefing is an essential component of any simulated learning experience [18]. Validity evidence, including reliability, for the DES has been gathered in previous studies [19], including by the present research team [20]. While the DES includes four subscales, one subscale, the Learning and Making Connections subscale was deemed appropriate for this evaluation. All items, except for overall satisfaction, were rated on a 5-point Likert scale from Strongly Disagree to Strongly Agree. Likert scale items were treated as pseudo-interval. Students were also asked to identify their program of study and the length of their program and to report the number of Virtu-WIL simulations they had completed. Two open-ended items were also included to capture the impact of VS on readiness for practice.: “*If the Virtu-WIL program increased your readiness for clinical practice, please share an example*” and, “*If appropriate, please provide an example of something new or different that you are doing in clinical practice after completing the Virtu-WIL program*.”

### Data analysis

Data were analyzed using frequency and descriptive statistics and ANOVA. All analyses were conducted in R (R Core Team, 2022) using the Psych [21] and dplyr packages [22]. Based on the unbalanced program sample sizes, all inferential statistical analysis, e.g., ANOVA, was conducted using a randomly stratified sub-sample with 60 data points from each group.

A preliminary check of the DES, and mini-PXI was conducted. The mini-PXI and DES were significantly correlated at *r* = .53. Internal reliability was computed for the mini-PXI and DES, with Cronbach’s alpha for both the DES and mini-PXI being .93. Cronbach’s alpha for both the Functional and Psychosocial subscales of the mini-PXI was .87 providing further evidence for the reliability of the survey items [23].

Seven members of the working group analyzed the two open-ended items, modifying a process described by Braun and Clarke [24]. A preliminary analysis was conducted to identify major themes, after which, all comments were read, coded, and then clustered under the major themes. Verbatim comments were extracted from the data to illustrate themes.

## Results

All 1715 (100%) students enrolled at the end of 2022 and beginning of 2023 in the Virtu-WIL program from 18 schools and four provinces responded to the survey. Over 60% provided feedback to the two open-ended items. Of the survey respondents, most were from NUR (1456; 84.9%), followed by PM (188; 10%) and MLT (71; 4.1%). The distribution by program was almost identical to the expected proportion of respondents based on enrollment in the program, indicating the current sample is representative of the population of participants completing the Virtu-WIL program. Participants reported being in programs ranging from 1 to 5 years in length, with most being in two (546, 31.8%) or three (722, 42.1%) year programs and the majority presently being in the first (790, 46.2%) or second (568, 33.2%) year of their studies. Students completed, on average, 3.4 (.96) virtual simulations and many repeated those simulations.

### Overall satisfaction and support

Overall, most respondents were satisfied or very satisfied (1455, 86.2%) with their Virtu-WIL experience. One hundred and twenty-nine (7.6%) were very dissatisfied, 25 (1.5%) were dissatisfied, while 80 (4.7%) were neither satisfied nor dissatisfied. Most (1336; 78.4%) participants reported that they had received the support they needed, while 113 (6.6%) felt they had not, and 256 (15.0%) did not think support was relevant to their Virtu-WIL experience. Of the 113 respondents who reported they had not received the support they needed, 17 (15.0%) reported being dissatisfied or very dissatisfied while the rest were satisfied or very satisfied (80; 70.7%), or neither satisfied nor dissatisfied (15;13.2%).

### Overall experience with the VS

Responses to three items that measured a sense of psychological safety, inclusion, and whether students would recommend the Virtu-WIL VSs to others were very positive. Mean scores for the total group were all higher than 4.4 out of a possible 5 (Table 1). The mean for recommending the program was somewhat lower for PM students (3.85).

**Table 1:** Overall experience with the VS, mean (SD), by total group and program

Item	All	NUR	MLT	PM
Psychologically safe learning experience.	4.65 .56	4.67 .56	4.63 .54	4.51 .58
Inclusive learning experience.	4.54 .65	4.58 .62	4.34 .74	4.28 .78
Recommend the program to peers and colleagues as a way to prepare for clinical practice	4.44 .80	4.52 .72	.4.35 .80	3.85 1.12

### Functionality and engagement

A major evaluation question was: Did the Virtu-WIL virtual simulations function well and engage students? Function and engagement were determined based on the results of the mini-PXI (Table 2). The mean total score for all groups on the mini-PXI was 45.5 out of a possible 55, indicating a very positive experience for most students. The mean for the Functional subscale items was 4.2 (SD = .76) out of a possible 5, suggesting students found the VS, for the most part, easy to use, audio and visually appealing and sufficiently challenging. The mean for the Psychosocial subscale items was 4.12 (SD = .72) out of a possible 5, indicating that students felt they had been immersed in the simulations and that the VS situations were meaningful to them. All items on the mini-PXI for the total group had mean scores of 4.0 or higher.

**Table 2:** Mini-PXI items, mean (SD), by total group and program

	All	NUR	MLT	PM
**Functional Subscale **				
I liked the look and feel of the Virtu-WIL virtual simulations.	4.23 .87	4.34 .77	4.1 .76	3.44 1.15
The virtual simulations were not too easy and not too hard to play	4.14 .85	4.23 .79	3.99 .80	3.51 1.1
It was easy to know how to perform actions in the virtual simulations.	4.02 1.02	4.18 .89	3.63 .99	2.94 1.3
The goals of the Virtu-WIL virtual simulations were clear to me	4.34 .81	4.44 .69	4.15 .60	3.58 1.19
The virtual simulations gave me clear feedback on my progress toward the goals	4.21 .88	4.31 .77	4.1 .70	3.41 1.23
**Psychosocial Subscale **				
I felt free to play the virtual simulations in my own way	4.23 .90	4.33 .80	3.76 .99	3.57 1.2
I wanted to explore how the virtual simulations evolved	4.15 .82	4.2 .78	3.77 1.02	3.88 .94
I was fully focused on the Virtu-WIL virtual simulations	4.28 .75	4.32 .72	4.31 .71	3.99 .87
I felt I was good at playing the Virtu-WIL virtual simulations	4.18 .87	4.3 .76	3.77 .87	3.36 1.15
Playing the Virtu-WIL virtual simulations was meaningful to me	4.05 .86	4.12 .80	4.06 .65	3.48 1.11
I had a good time playing these Virtu-WIL virtual simulations	4.21 .81	4.28 .74	4.11 .80	3.69 1.09
**Total PXI (maximum score: 55)**	45.5 7.66	46.5 6.95	43.6 6.21	38.6 9.49
The simulation preparation and pre brief activities helped me make the most of my virtual simulation learning.	4.23 .78	4.30 .72	4.07 .82	3.74 .97
The Virtu-WIL virtual simulations scenarios reflected what I see in my clinical practice	4.16 .87	4.24 .81	4.03 .69	3.55 1.09

While results for the total group were very positive, they varied according to program. All item means on the mini-PXI for nursing were 4 out of a possible 5. The range for MLT was marginally lower, 3.63–4.31, and lower still for PM, 2.94–3.99. The item with the lowest score for both MLT and PM was, *It was easy to know how to perform actions in the virtual simulations.* Statistically significant differences were identified on the mini-PXI between PM and NUR and MLT on the Functional Subscale *F*(2,113) = 18.6. *p* < .001 and Psychosocial Subscale *F*(2,116) = 8.03. *p* < .001 and total scores *F*(2,114) = 14.8. *p* < .001 Post hoc analysis applying the Tukey correction indicated significant differences between PM and NUR *t*(177) = 5.80, *p* < .001 (mean difference = 1.47), and PM and MLT *t*(177) = 4.33, *p* < .001 (mean difference = 1.10), *p* <.001. No significant differences existed between MLT and NUR, *t*(177) = 1.47, *p* < .001 *p* = .31. This pattern, with the PM students reporting a lower score, also held with the item related to the effectiveness of prebriefing activities and the extent to which students perceived the VSs as reflecting what they see in clinical practice.

### Debriefing experience

We analyzed the data to determine if debriefing helped students learn and make connections between theory and practice. Students found the debriefing experience to be helpful, with an average DES item score of 4.28 (.78) out of a possible 5 (Table 3). Students reported that the debriefing deepened their learning and helped to clarify questions arising from the VS scenarios. The mean score for all items on the DES for the total group was 4.3 or greater. By group, PM had lower scores across the DES items as well on the total DES, the difference was marginally statistically significant (PM–MLT, *p* = .029, and PM–NUR, *p* = .027). The actual mean differences were relatively small (.36).

**Table 3:** Debriefing Experience Scale Items, mean (SD), by total group and program

Item	All	NUR	MLT	PM
Debriefing in the Virtu-WIL virtual simulations helped me to make connections in my learning.	4.41 .70	4.45 .66	4.43 .69	4.07 .87
Debriefing was helpful in making sense of the virtual simulation	4.43 .68	4.46 .66	4.34 .75	4.20 .71
Debriefing provided me with a learning opportunity.	4.44 .68	4.50 .64	4.32 .76	4.14 .76
Debriefing helped me to find meaning in the virtual simulation	4.32 .75	4.37 .73	4.19 .85	4.00 .80
My questions from the virtual simulation were answered by debriefing	4.33 .75	4.37 .73	4.25 .74	4.03 .81
Debriefing helped me become more aware of my role as a healthcare provider.	4.33 .74	4.41 .72	4.16 .78	3.73 .96
Debriefing helped me to clarify problems	4.32 .79	4.38 .72	4.26 .64	3.98 .85
**Average DES (maximum score: 5)**	4.37 .61	4.42 .58	4.28 .62	4.02 .68

### Impact on readiness for practice

Across programs, participants tended to agree that the VSs enhanced the knowledge and skills that they believe they will be able to use in practice and increased their sense of competence; all three practice items for the total group had mean scores of 4 or greater out of a possible 5 (Table 4). Results by program followed the pattern with PM rating each item lower. Some of the lowest mean scores on the survey existed for the impact on practice items for Paramedicine. Statistically significant differences were identified between PM and MLT, and PM and Nursing on all three items (*p*= <.001). No significant differences were identified between MLT and NUR.

**Table 4:** Impact of VS on practice items, mean (SD), by total group and program

Item	All	NUR	MLT	PM
I learned a lot from the Virtu-WIL Program	4.15 .86	4.26 .75	4.13 .79	3.36 1.17
Will be able use knowledge and skills in practice	4.34 .77	4.43 .66	4.34 .70	3.60 1.13
Feel more competent and read for practice	4.09 .87	4.18 .78	3.96 .84	3.41 1.19

## Results: open-ended items

### Impact of virtual simulations on competency and readiness for clinical practice

Three major themes were identified in the open-ended comments: development of essential skills, simulation-specific skills, and preparation for internship.

### Development of essential skills

Students noted that the VSs had helped them to develop skills that were essential for professional practice. These included clinical judgment, prioritization, delegation, problem-solving, communication (professional and therapeutic), and teamwork including collaboration, conflict management, and how to respond to bullying. Several students indicated that the VSs “helped improve my prioritization of care tasks" (NUR 1427) and clinical judgment. A NUR student noted that after the simulation, “I delegate more tasks and accept help from my colleagues” (NUR 622). Therapeutic communication skills were learned as demonstrated by one NUR student who noted, “the simulation taught me how to respond to a patient who feels mistreated or belittled" (NUR 986). A PM student indicated they changed, “how I approach patients or think about what to say to calm them down" (PM 1648). Communication skills needed in the team management of an acute situation were highlighted by a NUR student, “I started to announce loudly if I am administering a drug" (NUR 79). In addition, students demonstrated an improved understanding of team roles, one noted, “I learned that we can call the respiratory therapist when a patient is in respiratory distress" (NUR 895). Teamwork, conflict and bullying management skills were highlighted by a NUR student who indicated, “I liked the part about always making sure that everyone on our team knows their role, even if it’s a separation of duties, it’s always best to validate” (NUR 334). An MLT student indicated the VS “gave insight to the way a team could work together in the field” (MLT 63).

Lastly, other skills such as oral and written communication, time management, and multitasking were developed. Students noted that the VSs brought the theory they had learned in the classroom “alive”; they could better understand the theory when they saw it being applied by staff in the VSs. Students felt that by seeing concrete examples of the essential skills in action, those skills were sharpened.

### Simulation-specific skills

Students enumerated the practice skills they felt they had learned from the VSs that were specific to the VSs they had practiced. Examples included administering a puffer to a patient with asthma, caring for a patient with dementia, operating a PCA pump, treating anaphylaxis, managing a blood transfusion reaction, feeding a patient with dysphagia, and caring for a post-operative patient. These skills included assessment, treatment, and documentation for these clinical situations. The VSs helped develop these skills because the situations were realistic, and provided an opportunity to observe and practice. One NUR student noted the VSs, “allowed me to recognize my mistakes and correct them” (NUR 243). An MLT student said, “You are told over and over again why it is so important, but as a student it can be easy to brush over as a lot of time quality control is performed for you before lab due to time constraints. It was interesting to see how quality control plays a role in trouble shooting an [lab]analyzer” (MLT 46).

### Preparation for practice

Many students reported that the VSs had helped them prepare for their clinical practice, providing multiple explanations. The VSs “felt real” and helped students to “know what to expect” in the clinical setting and allowed students to “live these kinds of situations” that did not occur during their time in clinical. Students saw healthcare professionals playing out their roles which helped them in turn to picture their own future actions. Because students could better picture what they would actually be doing during clinical practice, many commented that they felt more confident regarding their emerging practice.

Many students gave concrete examples of how the VSs had affected their practice. A NUR student said when caring for patients with hearing loss they, “used to stand by the side” (NUR 84) and address the patient and now they “started going round and approaching him face to face" (NUR 84). Another noted, “I have learned that I should not hesitate to ask for help. I am trying to ask for help whenever I need it which makes me able to provide a safe care environment for the patients" (NUR 1509). A MLT student indicated they would be, “paying attention to small things—not overlooking important details” when doing lab analysis. While a PM student said they now ensure that “any patient who doesn’t wish to be transported by ambulance not only meets capacity but is also aware of the risks and has an alternate plan for care" (PM 1634).

It is important to note that while in the minority, not all students reported the VSs prepared them for practice. The most frequently cited reasons were that while they had completed the VSs, they had not yet had their clinical placement experience, or there had not been any opportunity, as yet, to see the impact of the VSs on their practice, or they had already experienced the situation in clinical. One MTL indicated that the VS did not impact their practice since “I already experienced these events in clinical, I don’t believe I’m doing anything that different right now” (MLT 59). That said, many were able to theorize or imagine how the VSs would benefit their future practice. One noted, “I haven’t started my internship yet, but I have a better idea about the decisions we will have to make during our work or internship” (NUR 207). Another factor that influenced students’ perceptions that the VS would not influence practice was that the VSs were not perceived as sufficiently challenging, Some students had come to their health program with considerable experience and noted the VSs were “a great refresher to some skills I have not utilized in a few years” (NUR 1260). In other cases, the VSs were not well-aligned with the curriculum. The VSs had been treated as a standalone activity and the impact on clinical practice was not apparent to students. The last factor was technology, and this was noted only in the PM group by several participants, with one saying, “but it was very frustrating since I kept running into technical issues, such as the simulation not picking up my microphone even though it said it was working and not knowing how all the tools work” (PM 1659).

## Discussion

This multi-site, pan-Canadian evaluation project involving three healthcare professions was conducted to measure the effectiveness of the Virtu-WIL program with a view to improving program quality. The project was large enough to provide a good sense of healthcare students’ experiences with VSs and the impact of VSs on learning and preparing students for practice. The evaluation consisted of a survey that included open-ended items. The multi-method approach worked well as the open-ended items shed light on, and supported, the quantitative data.

Most students were satisfied or very satisfied with their VS experience and most received the support they needed. Interestingly, of the relatively few who reported not receiving sufficient support, most described their experience as satisfactory or very satisfactory. This finding suggests that most Virtu-WIL VSs are relatively easy for students to use independently. Lower satisfaction scores were often attributed to technical problems with the VSs, particularly for the PM students, a finding reported in numerous earlier studies [25, 26] and one which points to the need for extensive testing before deploying a VS.

Regarding the overall student experience, responses to the three items that measured a sense of psychological safety, inclusion and whether students would recommend the VSs to their peers were very positive from all three program groups. This suggests that the way the VSs were designed and implemented contributed to a positive learning experience. The mini-PXI was used to measure VS functionality and student engagement and the mean score for all groups on this measure was 82/100 with somewhat lower scores for PM. This indicates that students found the VSs easy to use, esthetically and intrinsically appealing, sufficiently challenging, and helpful for learning. An important contributor to learning was that the students perceived the VSs as providing realistic experiences, a principle that is widely accepted as a necessity in healthcare simulation [27]. Students felt immersed in the simulations and the content/situations were meaningful to them; both of which are important factors that influence learning [6, 26]. As fidelity appears to play such an important role in learning, further research on its role in student engagement and learning and VS design is needed [28].

Most students reported a positive debriefing experience as it helped them to learn and to make sense of the VS scenario. While the total score for PM students was somewhat lower, the actual difference in mean scores was very small. The open-ended items suggest that the lower scores for PM students was related to the level of VS availability, the limited number of VSs for the PM students to choose from, and provincial practice differences. Lack of PM students’ prior clinical experience could also have made a difference to their perception of realism and the impact of the VSs on their learning [27]. That said, their survey scores were still quite positive, and the majority would recommend the VSs to their peers. The open-ended item responses from some NUR students pointed to the same issue; those with previous clinical experience found some of the VSs insufficiently challenging and viewed the VSs as a refresher. To maximize the effectiveness of the VSs, they should be embedded in the curriculum with careful consideration given to that placement [9]. The VSs need to align with content and course objectives [29] and not be either too simple or too complex [30, 31]. 

The need for VSs to align with curriculum is tied to another major finding: VS can play an important role in preparing students for clinical practice. We found, across programs, that playing a VS that reflects clinical practice and that students see as useful in enhancing their skills, strongly contributes to their perceptions of readiness for practice. Again, the open-ended items shed light on this finding; the VSs provided an opportunity for students to see their professional roles in action and to take on that role. Students had the chance to think things through, make mistakes, and learn from their mistakes in a safe environment. Research is emerging that giving students the opportunity to practice and fail in low stakes situations contributes to learning [32]. In addition, these students, some of whom had not yet been to clinical, found it invaluable to see a clinical scenario unfold and to see how health professionals managed the situation. Again, careful consideration regarding curriculum integration is essential to give students the opportunity to practice what they have learned in the VS and the timing or sequencing of VS needs further study [5, 33]. Ideally, students would have the opportunity to practice at several points in their program.

A recent meta-analysis [34] demonstrated that VS can significantly improve clinical reasoning and performance in students. However, because clinicians draw on a body of knowledge when making decisions for individuals, there is an art to making those decisions. While VSs provide students with rich, standardized scenarios for practice, currently, the tacit knowledge of clinical practice can only emerge through discussions in the debriefing. A major finding from this study is that the use of simulation pedagogy is key to achieving those outcomes, uncovering tacit knowledge, and promoting readiness for practice. Students in this study rated the prebriefing and debriefing activities as essential to their learning, a finding that has been reported in earlier studies [35–37]. Anyone facilitating VS needs training in this process [38].

## Limitations

One limitation of the study is that we did not collect information on students’ pre-program clinical experience such as previous educational qualifications and life experience. Prior experience may have influenced students’ perceptions of the realism of the VS and therefore influenced learning and satisfaction outcomes. While most participants were from nursing, the distribution of students is representative of the population of students participating in the Virtu-WIL program. Strengths of the study were the large, multi-site, cross-provincial sample, and the use of validated tools in the survey subscales.

## Conclusion

The pandemic and the pressures to graduate larger numbers of healthcare professionals in response to the health human resource crisis have created major challenges in providing quality work-integrated learning experiences for students. VS has emerged as a learning modality that can help students develop clinical reasoning, problem-solving, and other key employability skills. The results of this study indicate that students found VSs engaging, helping them learn and prepare for practice. A key finding was that it is not sufficient to simply add VSs to the curriculum, careful planning, and simulation pedagogy are essential. Further research is needed regarding many aspects of VS and, in particular, effective curriculum integration strategies and the timing or sequencing of VS to best prepare students for practice. As the quality of the VS experience improves for students, VS may be used as an effective way to decrease the in-person clinical hours needed to be ready for practice.

### Supplementary Information

**Additional file 1.**

## Data Availability

Access to aggregate data and other analysis completed available on request. Individual data sets are not available.

## Abbreviations

NUR: Nursing
MLT: Medical Laboratory Technologist
PM: Paramedic
Virtu-WIL: Virtual work-integrated learning
VS: Virtual simulation
