# Supplementary material for: Virtual simulation in healthcare education: a multi-professional, pan-Canadian evaluation
Source: Adv Simul (Lond). 2024 Jan 10;9:3. doi: 10.1186/s41077-023-00276-x (PMC10782638; doi:10.1186/s41077-023-00276-x)
Supplement: Supplementary file 1 — Additional file 1. [file 41077_2023_276_MOESM1_ESM.docx]

**Supplemental Materials**

**Sample Demographics**

**Supplemental Table 1.** Frequency of respondents by School and Province representing Count and Percent of total sample

|  | Province | | | |
| --- | --- | --- | --- | --- |
| School | Ontario | Quebec | Alberta | British Columbia |
| Seneca College | 179 (10.4%) |  |  |  |
| Nipissing University | 159 (9.3%) |  |  |  |
| Fanshawe College | 113 (6.6%) |  |  |  |
| Loyalist College | 103 (6.0%) |  |  |  |
| George Brown College | 89 (5.2%) |  |  |  |
| Algonquin College | 42 (2.4%) |  |  |  |
| Fleming College | 24 (1.4%) |  |  |  |
| Toronto Metropolitan University | 6 (0.3%) |  |  |  |
| Collège Boréal | 6 (0.3%) |  |  |  |
| Université de Montréal |  | 577 (33.6%) |  |  |
| Cégep Édouard-Montpetit |  | 162 (9.4%) |  |  |
| Cégep André-Laurendeau |  | 35 (2.0%) |  |  |
| Cégep de Chicoutimi |  | 29 (1.7%) |  |  |
| Cégep de Sherbrooke |  | 20 (1.2%) |  |  |
| Northern Alberta Institute of Technology |  |  | 75 (4.4%) |  |
| Southern Alberta Institute of Technology |  |  | 20 (1.2%) |  |
| Lethbridge College |  |  | 3 (0.2%) |  |
| Camosun College |  |  |  | 73 (4.3%) |
| Count | 721 (42.0%) | 823 (48.0%) | 98 (5.7%) | 73 (4.3%) |

**Supplemental Table 2.** Proportion of respondents compared to the expected

|  | Count (% of Total) | | |
| --- | --- | --- | --- |
| Program | 2022-23 | 2023-24* | 2023** |
| Nursing | 1464 (85%) | 3895 (86%) | 1456 (84.9%) |
| Paramedicine | 188 (11%) | 451 (10%) | 188 (11.0%) |
| MLT | 71 (4%) | 167 (4%) | 71 (4.1%) |

*Expected total of Virtu-Wil participants at the conclusion of the program

**Actual as of time of reporting

**Supplemental Table 3.** Frequencies of sample Program Length and Year

| Program | Count | Program | Count |
| --- | --- | --- | --- |
| Length | (% of Total) | Year* | (% of Total) |
| 1 | 155 (9.0) | 1 | 790 (46.2) |
| 2 | 546 (31.8) | 2 | 568 (33.2) |
| 3 | 722 (42.1) | 3 | 311 (18.2) |
| 4 | 257 (15.0) | 4 | 34 (2.0) |
| 5 | 35 (2.0) | 5 | 7 (0.4) |

*n missing = 5

**Supplemental Table 4.** Frequencies of sample Program Length and Year by Program

|  |  | Program Length | | Current Year* | |
| --- | --- | --- | --- | --- | --- |
| Year | Program | Counts | % of Total | Counts | % of Total |
| 1 | Nursing | 88 | 5.1 % | 639 | 37.4 % |
|  | Medical Laboratory Technology | 23 | 1.3 % | 44 | 2.6 % |
|  | Paramedicine | 44 | 2.6 % | 107 | 6.3 % |
| 2 | Nursing | 371 | 21.6 % | 471 | 27.5 % |
|  | Medical Laboratory Technology | 39 | 2.3 % | 21 | 1.2 % |
|  | Paramedicine | 136 | 7.9 % | 76 | 4.4 % |
| 3 | Nursing | 707 | 41.2 % | 300 | 17.5 % |
|  | Medical Laboratory Technology | 8 | 0.5 % | 6 | 0.4 % |
|  | Paramedicine | 7 | 0.4 % | 5 | 0.3 % |
| 4 | Nursing | 255 | 14.9 % | 34 | 2.0 % |
|  | Medical Laboratory Technology | 1 | 0.1 % | 0 | 0.0 % |
|  | Paramedicine | 1 | 0.1 % | 0 | 0.0 % |
| 5 | Nursing | 35 | 2.0 % | 7 | 0.4 % |
|  | Medical Laboratory Technology | 0 | 0.0 % | 0 | 0.0 % |
|  | Paramedicine | 0 | 0.0 % | 0 | 0.0 % |

*n missing = 5

**Overall VIRTU-WIL Program Experience**

**Supplemental Table 6.** Frequencies for overall satisfaction by total sample and stratified sample

|  |  | Total | | Stratified | |
| --- | --- | --- | --- | --- | --- |
| Satisfaction | Program | Counts | % Total | Counts | % Total |
| Very Dissatisfied | Nursing | 123 | 7.3 % | 2 | 1.1 % |
|  | MLT | 2 | 0.1 % | 2 | 1.1 % |
|  | Paramedicine | 4 | 0.2 % | 1 | 0.6 % |
| Dissatisfied | Nursing | 7 | 0.4 % | 1 | 0.6 % |
|  | MLT | 1 | 0.1 % | 1 | 0.6 % |
|  | Paramedicine | 17 | 1.0 % | 6 | 3.4 % |
| Neither satisfied nor dissatisfied | Nursing | 48 | 2.8 % | 4 | 2.2 % |
|  | MLT | 2 | 0.1 % | 2 | 1.1 % |
|  | Paramedicine | 30 | 1.8 % | 9 | 5.0 % |
| Satisfied | Nursing | 705 | 41.7 % | 37 | 20.7 % |
|  | MLT | 40 | 2.4 % | 33 | 17.9 % |
|  | Paramedicine | 97 | 5.7 % | 34 | 19.0 % |
| Very Satisfied | Nursing | 550 | 32.6 % | 14 | 8.4 % |
|  | MLT | 25 | 1.5 % | 23 | 12.8 % |
|  | Paramedicine | 38 | 2.2 % | 10 | 5.6 % |

**Supplemental Table 7.** Frequencies of Support Received by total sample and stratified sample

|  |  | Total | | Stratified | |
| --- | --- | --- | --- | --- | --- |
| Received  Support | Program | Counts | % Total | Counts | % Total |
| Yes | Nurse | 1132 | 66.4 % | 46 | 25.6 % |
|  | MLT | 61 | 3.6 % | 53 | 29.4 % |
|  | Para | 143 | 8.4 % | 43 | 23.9 % |
| No | Nurse | 91 | 5.3 % | 4 | 2.2 % |
|  | MLT | 0 | 0.0 % | 0 | 0.0 % |
|  | Para | 22 | 1.3 % | 10 | 5.3 % |
| N/A | Nurse | 224 | 13.1 % | 10 | 5.6 % |
|  | MLT | 9 | 0.5 % | 7 | 3.9 % |
|  | Para | 23 | 1.3 % | 7 | 3.9 % |

**Supplemental Table 8.** Descriptive statistics for measures of overall experience by total sample, stratified sample, and program

|  |  | PsychSafe | | | | Inclusive | | | | Recommend | | | |
| --- | --- | --- | --- | --- | --- | --- | --- | --- | --- | --- | --- | --- | --- |
|  | Sample | All | Nurse | MLT | Para | All | Nurse | MLT | Para | All | Nurse | MLT | Para |
| Mean | Total | 4.65 | 4.67 | 4.63 | 4.51 | 4.54 | 4.58 | 4.34 | 4.28 | 4.44 | 4.52 | 4.35 | 3.85 |
|  | Strat | 4.59 | 4.68 | 4.63 | 4.45 | 4.35 | 4.54 | 4.33 | 3.77 | 4.16 | 4.37 | 4.33 | 3.77 |
| Median | Total | 5 | 5 | 5 | 5 | 5 | 5 | 4 | 4 | 5 | 5 | 4 | 4 |
|  | Strat | 5 | 5 | 5 | 4 | 4 | 5 | 5 | 4 | 4 | 5 | 5 | 4 |
| SD | Total | 0.56 | 0.561 | 0.54 | 0.581 | 0.65 | 0.62 | 0.74 | 0.78 | 0.8 | 0.72 | 0.8 | 1.12 |
|  | Strat | 0.538 | 0.51 | 0.55 | 0.54 | 0.74 | 0.54 | 0.77 | 0.85 | 0.959 | 0.72 | 0.82 | 1.174 |

PsychSafe: *Participating in the Virtu-WIL program was a psychologically safe learning experience*

*Inclusive: Participating in the Virtu-WIL program was an inclusive learning experience*

*Recommend: I would recommend the Virtu-WIL program to my peers and colleagues as a way of preparing for clinical practice*

**Experiences with the** **Virtu-Wil virtual simulations in the Virtu-Wil Program**

**Supplemental Table 9.** Descriptive statistics for the utility of preparation and pre-briefing activities by total and stratified sample and program

|  | Mean | | | | Median | | | | SD | | | |
| --- | --- | --- | --- | --- | --- | --- | --- | --- | --- | --- | --- | --- |
|  | All | Nurse | MLT | Para | All | Nurse | MLT | Para | All | Nurse | MLT | Para |
| Total | 4.23 | 4.30 | 4.07 | 3.74 | 4 | 4 | 4 | 4 | 0.78 | 0.72 | 0.82 | 0.97 |
| Stratified | 3.96 | 4.10 | 4.11 | 3.66 | 4 | 4 | 4 | 4 | 0.91 | 0.74 | 0.82 | 1.08 |

**Supplemental Table 10.** Descriptive statistics of the mini-PXI components by total and stratified sample and program

|  |  | Mean | | | | Median | | | | SD | | | |
| --- | --- | --- | --- | --- | --- | --- | --- | --- | --- | --- | --- | --- | --- |
| Scale* | Sample | All | Nurse | MLT | Para | All | Nurse | MLT | Para | All | Nurse | MLT | Para |
| F-PXI | Total | 4.16 | 4.27 | 3.99 | 3.36 | 4.2 | 4.4 | 4 | 3.6 | 0.76 | 0.672 | 0.563 | 0.981 |
|  | Strat | 3.8 | 4.18 | 3.9 | 3.23 | 4 | 4.2 | 4 | 3.4 | 0.88 | 0.61 | 0.59 | 1.05 |
| Raw-F-PXI | Total | 20.8 | 21.4 | 20 | 16.8 | 21 | 22 | 20 | 18 | 3.82 | 3.36 | 2.81 | 4.9 |
|  | Strat | 19 | 20.9 | 19.9 | 16.1 | 20 | 21 | 20 | 17 | 4.39 | 3.03 | 2.97 | 5.25 |
| PS-PXI | Total | 4.12 | 4.19 | 3.95 | 3.64 | 4.17 | 4.17 | 4 | 3.83 | 0.72 | 0.679 | 0.624 | 0.85 |
|  | Strat | 3.9 | 4.13 | 3.95 | 3.62 | 4 | 4 | 4 | 3.8 | 0.72 | 0.58 | 0.66 | 0.82 |
| Raw-PS-PXI | Total | 24.7 | 25.2 | 23.7 | 21.8 | 25 | 25 | 24 | 23 | 4.32 | 4.08 | 3.74 | 5.1 |
|  | Strat | 23.4 | 24.8 | 23.7 | 21.7 | 24 | 24 | 24 | 23 | 4.33 | 3.45 | 3.95 | 4.92 |
| Ttl-PXI | Total | 8.28 | 8.46 | 7.94 | 6.99 | 8.37 | 8.53 | 8 | 7.37 | 1.4 | 1.26 | 1.12 | 1.74 |
|  | Strat | 7.7 | 8.31 | 7.94 | 6.84 | 8 | 8.08 | 8 | 7.3 | 1.52 | 1.06 | 1.19 | 1.8 |
| Raw-T-PXI | Total | 45.5 | 46.5 | 43.6 | 38.6 | 46 | 47 | 44 | 41 | 7.66 | 6.95 | 6.21 | 9.49 |
|  | Strat | 42.4 | 45.7 | 43.6 | 37.8 | 44 | 44.5 | 44 | 40.5 | 8.27 | 5.84 | 6.58 | 9.8 |

*F-PXI = Functional PXI, Scoring is Total/# Items

Raw-F-PXI = Raw Functional PXI, Scoring is Absolute Score on F-PXI, max = 25

PS-PXI = Psycho-Social PXI, Scoring is Total/# Items

Raw-PS-PXI = Raw Psycho-Social PXI, Scoring is Absolute Score on PS-PXI, max = 30

Ttl-PXI = Total PXI, Scoring is F-PXI+PS-PXI

Raw-T-PXI = Raw Total PXI, Scoring is Raw-F-PXI+Raw-PS-PXI, max = 55

**Supplemental Table 11.** Descriptive statistics for Items of the Functional-PXI by total sample, stratified sample, and program

|  |  | Look/Feel | | Difficulty | | HowtoPlay | | GoalsClear | | Feedback | |
| --- | --- | --- | --- | --- | --- | --- | --- | --- | --- | --- | --- |
|  | Program | Total | Strat | Total | Strat | Total | Strat | Total | Strat | Total | Strat |
| Mean | Nurse | 4.34 | 4.22 | 4.23 | 4.13 | 4.18 | 4.17 | 4.44 | 4.37 | 4.31 | 4.17 |
|  | MLT | 4.1 | 4.1 | 3.99 | 3.98 | 3.63 | 3.62 | 4.15 | 4.17 | 4.1 | 4.08 |
|  | Para | 3.44 | 3.33 | 3.51 | 3.37 | 2.94 | 2.87 | 3.58 | 3.46 | 3.41 | 3.29 |
| Median | Nurse | 4 | 4 | 4 | 4 | 4 | 4 | 5 | 4 | 4 | 4 |
|  | MLT | 4 | 4 | 4 | 4 | 4 | 4 | 4 | 4 | 4 | 4 |
|  | Para | 4 | 4 | 4 | 4 | 3 | 3 | 4 | 4 | 4 | 4 |
| SD | Nurse | 0.77 | 0.74 | 0.79 | 0.83 | 0.89 | 0.79 | 0.69 | 0.55 | 0.77 | 0.87 |
|  | MLT | 0.76 | 0.75 | 0.8 | 0.87 | 0.99 | 1.04 | 0.6 | 0.62 | 0.7 | 0.72 |
|  | Para | 1.15 | 1.14 | 1.1 | 1.14 | 1.3 | 1.28 | 1.19 | 1.25 | 1.23 | 1.29 |
|  | Mean | 4.23 | 3.88 | 4.14 | 3.83 | 4.02 | 3.55 | 4.34 | 4 | 4.21 | 3.85 |
| All | Median | 4 | 4 | 4 | 4 | 4 | 4 | 4 | 4 | 4 | 4 |
|  | SD | 0.87 | 0.98 | 0.85 | 1.01 | 1.02 | 1.18 | 0.81 | 0.94 | 0.88 | 1.06 |

**Supplemental Table 12.** Descriptive statistics for Items of the Psycho-Social-PXI by total sample, stratified sample, and program

|  |  | FreePlay | | ExploreSims | | FullFocus | | GoodatPlaying | | Meaningful | | GoodTime | |
| --- | --- | --- | --- | --- | --- | --- | --- | --- | --- | --- | --- | --- | --- |
|  | Program | Total | Strat | Total | Strat | Total | Strat | Total | Strat | Total | Strat | Total | Strat |
| Mean | Nurse | 4.33 | 4.2 | 4.2 | 4.07 | 4.32 | 4.3 | 4.3 | 4.27 | 4.12 | 4.0 | 4.28 | 4.22 |
|  | MLT | 3.76 | 3.77 | 3.77 | 3.81 | 4.31 | 4.33 | 3.77 | 3.75 | 4.06 | 4.05 | 4.11 | 4.12 |
|  | Para | 3.57 | 3.37 | 3.88 | 3.83 | 3.99 | 4.02 | 3.36 | 3.32 | 3.48 | 3.5 | 3.69 | 3.7 |
| Median | Nurse | 4 | 4 | 4 | 4 | 4 | 4 | 4 | 4 | 4 | 4 | 4 | 4 |
|  | MLT | 4 | 4 | 4 | 4 | 4 | 4 | 4 | 4 | 4 | 4 | 4 | 4 |
|  | Para | 4 | 4 | 4 | 4 | 4 | 4 | 4 | 3.5 | 4 | 4 | 4 | 4 |
| SD | Nurse | 0.8 | 0.82 | 0.78 | 0.86 | 0.72 | 0.65 | 0.76 | 0.66 | 0.8 | 0.87 | 0.74 | 0.74 |
|  | MLT | 0.99 | 0.98 | 1.02 | 1.05 | 0.71 | 0.75 | 0.87 | 0.9 | 0.65 | 0.7 | 0.8 | 0.85 |
|  | Para | 1.2 | 1.3 | 0.94 | 0.91 | 0.87 | 0.81 | 1.15 | 1.16 | 1.11 | 1.08 | 1.09 | 1.08 |
| All | Mean | 4.23 | 3.78 | 4.15 | 3.9 | 4.28 | 4.22 | 4.18 | 3.78 | 4.05 | 3.85 | 4.21 | 4.01 |
|  | Median | 4 | 4 | 4 | 4 | 4 | 4 | 4 | 4 | 4 | 4 | 4 | 4 |
|  | SD | 0.9 | 1.1 | 0.82 | 0.95 | 0.75 | 0.75 | 0.87 | 1 | 0.86 | 0.93 | 0.81 | 0.92 |

**Supplemental Table 13.** Significant differences and mean differences between programs on the mini-PXI for the stratified sample

|  | Scale* | MLT | Nurse |
| --- | --- | --- | --- |
| Nurse | F-PXI | p = .185, 0.19^a^ |  |
|  |  |  |  |
|  | PS-PXI | p = 0.24, 0.19 |  |
|  |  |  |  |
|  | T-PXI | p = .18, 0.37 |  |
|  |  |  |  |
| Para | F-PXI | p < .001, 0.76 | p < .001, 0.95 |
|  | PS-PXI | p = .04, 0.34 | p < .001, 0.52 |
|  | T-PXI | p < .001, 1.1 | p < .001, 1.5 |

*F-PXI = Functional PXI

PS-PXI = Psycho-Social PXI

T-PXI = Total PXI

^a^ Mean Difference

**Debriefing Experience**

**Supplemental Table 14.** Descriptive Statistics for the DES by total sample and stratified sample

|  |  | Debriefing Evaluation Scale | | | |
| --- | --- | --- | --- | --- | --- |
|  | Sample | Total | Nurse | MLT | Para |
| Mean | Total | 4.37 | 4.42 | 4.28 | 4.02 |
|  | Strat | 4.21 | 4.32 | 4.34 | 3.99 |
| SD | Total | .61 | 0.58 | 0.62 | 0.68 |
|  | Strat | 0.66 | 0.60 | 0.61 | 0.71 |
| Median | Total | 4.57 | 4.57 | 4.36 | 4 |
|  | Strat | 4.14 | 4.29 | 4.43 | 4 |

**Supplemental Table 15.** Descriptive Statistics for Items of the DES by total sample, stratified sample and program

|  |  | Connect | | Sense | | Learn | | Meaning | | AnswrdQs | | RoleAware | | Clarify | |
| --- | --- | --- | --- | --- | --- | --- | --- | --- | --- | --- | --- | --- | --- | --- | --- |
|  | Program | Total | Strat | Total | Strat | Total | Strat | Total | Strat | Total | Strat | Total | Strat | Total | Strat |
| Mean | Nurse | 4.45 | 4.32 | 4.45 | 4.33 | 4.49 | 4.36 | 4.36 | 4.19 | 4.37 | 4.29 | 4.4 | 4.31 | 4.38 | 4.41 |
|  | MLT | 4.37 | 4.37 | 4.31 | 4.38 | 4.3 | 4.37 | 4.15 | 4.2 | 4.25 | 4.33 | 4.14 | 4.2 | 4.26 | 4.32 |
|  | Para | 4.05 | 4.03 | 4.18 | 4.22 | 4.13 | 4.03 | 3.99 | 3.98 | 4.03 | 4.07 | 3.73 | 3.74 | 3.98 | 3.83 |
| Median | Nurse | 5 | 4 | 5 | 4 | 5 | 4 | 4 | 4 | 4 | 4 | 5 | 4 | 4 | 4 |
|  | MLT | 4 | 5 | 4 | 4.5 | 4 | 5 | 4 | 4 | 4 | 4 | 4 | 4 | 4 | 4 |
|  | Para | 4 | 4 | 4 | 4 | 4 | 4 | 4 | 4 | 4 | 4 | 4 | 4 | 4 | 4 |
| SD | Nurse | 0.66 | 0.7 | 0.66 | 0.68 | 0.65 | 0.74 | 0.73 | 0.75 | 0.72 | 0.79 | 0.73 | 0.7 | 0.72 | 0.53 |
|  | MLT | 0.76 | 0.8 | 0.75 | 0.74 | 0.77 | 0.74 | 0.86 | 0.88 | 0.74 | 0.66 | 0.79 | 0.81 | 0.63 | 0.63 |
|  | Para | 0.88 | 1 | 0.73 | 0.7 | 0.76 | 0.81 | 0.8 | 0.78 | 0.81 | 0.79 | 0.96 | 0.97 | 0.86 | 1.01 |
| All | Mean | 4.41 | 4.27 | 4.43 | 4.33 | 4.44 | 4.26 | 4.33 | 4.15 | 4.33 | 4.23 | 4.32 | 4.09 | 4.32 | 4.19 |
|  | Median | 4 | 4 | 5 | 4 | 5 | 4 | 4 | 4 | 4 | 4 | 4 | 4 | 4 | 4 |
|  | SD | 0.7 | 0.85 | 0.68 | 0.71 | 0.68 | 0.78 | 0.75 | 0.81 | 0.74 | 0.76 | 0.79 | 0.86 | 0.79 | 0.79 |

**Impact on Practice**

**Supplemental Table 16.** Descriptive Statistics for impact on practice by total and stratified sample and program

|  |  | Mean | | | | Median | | | | SD | | | |
| --- | --- | --- | --- | --- | --- | --- | --- | --- | --- | --- | --- | --- | --- |
|  |  | All | Nurse | MLT | Para | All | Nurse | MLT | Para | All | Nurse | MLT | Para |
| Learn | Total | 4.15 | 4.26 | 4.13 | 3.36 | 4 | 4 | 4 | 4 | 0.86 | 0.75 | 0.79 | 1.17 |
|  | Strat | 3.86 | 4.22 | 4.13 | 3.22 | 4 | 4 | 4 | 4 | 1.06 | 0.77 | 0.85 | 1.21 |
| UseK&S | Total | 4.34 | 4.43 | 4.34 | 3.6 | 4 | 5 | 4 | 4 | 0.77 | 0.66 | 0.7 | 1.13 |
|  | Strat | 4.04 | 4.32 | 4.4 | 3.41 | 4 | 4 | 4 | 4 | 1.03 | 0.68 | 0.72 | 1.29 |
| Comp | Total | 4.09 | 4.18 | 3.96 | 3.41 | 4 | 4 | 4 | 4 | 0.87 | 0.78 | 0.84 | 1.19 |
|  | Strat | 3.76 | 4.02 | 3.97 | 3.29 | 4 | 4 | 4 | 4 | 1.06 | 0.81 | 0.88 | 1.29 |

**Supplemental Table 17.** Significant differences and mean differences between programs on the impact on practice items for the stratified sample

|  | Scale* | MLT | Nurse |
| --- | --- | --- | --- |
| Nurse | LearnLot | p = .83, 0.08^a^ |  |
|  |  |  |  |
|  | UseK&S | p = 0.79, 0.08 |  |
|  |  |  |  |
|  | Comp | p = .94, 0.05 |  |
|  |  |  |  |
| Para | LearnLot | p < .001, 0.91 | p < .001, 0.99 |
|  | UseK&S | p < .001, 0.99 | p < .001, 0.91 |
|  | Comp | p = .003, .68 | p < .001, 0.73 |

*LearnLot = I learned a lot from the Virtu-WIL Program

UseK&S = I believe I will be able to use the knowledge and skills I developed with the Virtu-WIL Program in my clinical practice/practicum.

Comp = I feel more competent and ready for clinical practice/practicum having participated in the Virtu-WIL Program

^a^ Mean Difference
